# Herniotomy

**Published:** 1886-06-20

**Authors:** C. J. Carter

**Affiliations:** Georgia


### HERNIOTOMY.

BY C. J. CARTER, M. D., OF GEORGIA.

It being my first attempt to report anything in my much-prized profession, hope all due allowance will be made.

On the night of March 7, 1886, was called to Edmond S——, aged about thirty-five. Found him suffering with strangulated hernia. As good fortune would have it my preceptor was spending the night with me, and consented to see the case with me on the morning of the 8th. We tried taxis perseveringly, cold applications, tartar emetic, opium, etc., without avail. On the evening of the 8th, I secured the services of my brother, who had just graduated, and then tried chloroform ; still failed. It was then getting so late I could not think of operating, so ventured to postpone it. I am sure I should have operated while he was under the anæsthetic, but, as I had never performed the operation, I wanted the best light possible. I gave opium, and decided to call next morniug. Called on the morning of the 9th, determined to operate, but, on arrival, found my needles misplaced, and was delayed till 3 p. m., when we began the administration of the anæsthetic; having a very stout man to deal with, we were delayed in getting him thoroughly anæsthetized. It being a case of external oblique, I made my incision near the central point, between symphisis pubis and superior spinous process of the ilium; first making it two inches long, but finding it not large enough, enlarged it to near four inches before being able to return the bowel, and on returning the bowel, we found about six ounces of sanious fluid in the scrotum, and about four inches of strictured bowel, purple almost to blackness. By protecting the opening in the abdomen, my assistant pressed the fluids out of the scrotum while the bowel was being returned. When they were returned, I made two stitches and dressed with bandage and antiseptic applications. As soon as he rallied from the chloroform sufficiently I gave him 2 ozs. whisky and 15 grains of quinine. Would say that I had not as much as an ounce of hemorrhage; did not have to ligate or compress an artery. Left with instructions to give 15 or 20 drops of tinct. opii deod. and 5 grains quinine every three hours, except when asleep; not to be moved till so ordered.

Saw him on the 10th; continued opium and quinine. Saw him again on the 11th; continued quinine, but less opium—only gave it when required. Saw him again on the 12th; had no fever yet, but continued quinine. 13th, still all going well. 14th, slight discharge from incision. 15th, discharge pretty free. 16th, discharge ceased. Saw him every day till the 25th.

As he had been kept on a light diet with no solids, his bowels were locked until the 26th, when I withheld all opiates, and they moved without a cathartic of any kind. Ordered a truss and put it on him on the 28th, when he got up and walked. I gave him from 40 to 60 grains quinine per day till he had taken about 460 grains. He went to plowing soon after, and I have not seen him since.

I think I should have operated sooner, but was overpersuaded by older heads, and should I encounter another similar case would operate during the first twenty-four hours. Did I give him more quinine than was necessary, and was I correct in keeping the bowels locked so long? Why could I have made an incision as large as the above and not had a greater hemorrhage?

[IN reply to questions asked, will state that so large doses of quinine would seem to have been unnecessary. It is well to keep the bowels locked for a *few* days, especially in cases where gangrene or disorganization in the bowel is feared. There would seem to be no necessity for confining the bowels for so long a time as was done in this case. The length of the incision would not materially increase the hemorrhage where no artery of size was cut. The hemorrhage in this operation is seldom troublesome, unless the epigastric artery is wounded in dividing the strictured point. Caution in the young operator is commendable, even though it be carried a little too far ; and we congratulate our young professional friend on the success of this his first operation for hernia.—EDITOR.]

